# Subsea Cable Tracking by Autonomous Underwater Vehicle with Magnetic Sensing Guidance

**DOI:** 10.3390/s16081335

**Published:** 2016-08-20

**Authors:** Xianbo Xiang, Caoyang Yu, Zemin Niu, Qin Zhang

**Affiliations:** 1 School of Naval Architecture and Ocean Engineering, Huazhong University of Science and Technology, 1037, Luoyu Road, Wuhan 430074, China; ycyhust@hust.edu.cn (C.Y.); niuzemin@hust.edu.cn (Z.N.); 2 State Key Lab of Digital Manufacturing, Equipment and Technology, Huazhong University of Science and Technology, 1037, Luoyu Road, Wuhan 430074, China; qin.zhang@hust.edu.cn

**Keywords:** cable tracking, under-actuated AUV, magnetic sensing, path following, LOS guidance

## Abstract

The changes of the seabed environment caused by a natural disaster or human activities dramatically affect the life span of the subsea buried cable. It is essential to track the cable route in order to inspect the condition of the buried cable and protect its surviving seabed environment. The magnetic sensor is instrumental in guiding the remotely-operated vehicle (ROV) to track and inspect the buried cable underseas. In this paper, a novel framework integrating the underwater cable localization method with the magnetic guidance and control algorithm is proposed, in order to enable the automatic cable tracking by a three-degrees-of-freedom (3-DOF) under-actuated autonomous underwater vehicle (AUV) without human beings in the loop. The work relies on the passive magnetic sensing method to localize the subsea cable by using two tri-axial magnetometers, and a new analytic formulation is presented to compute the heading deviation, horizontal offset and buried depth of the cable. With the magnetic localization, the cable tracking and inspection mission is elaborately constructed as a straight-line path following control problem in the horizontal plane. A dedicated magnetic line-of-sight (LOS) guidance is built based on the relative geometric relationship between the vehicle and the cable, and the feedback linearizing technique is adopted to design a simplified cable tracking controller considering the side-slip effects, such that the under-actuated vehicle is able to move towards the subsea cable and then inspect its buried environment, which further guides the environmental protection of the cable by setting prohibited fishing/anchoring zones and increasing the buried depth. Finally, numerical simulation results show the effectiveness of the proposed magnetic guidance and control algorithm on the envisioned subsea cable tracking and the potential protection of the seabed environment along the cable route.

## 1. Introduction

Since the first underwater optical fiber cable came out in 1985, underwater cable systems have been extensively constructed worldwide and have played an important role in the field of cross-continental communications with the advantages of large capacity, high reliability and excellent communication quality. Yet, the underwater telecommunication cables are prone to suffer from potentially hazardous situations or environmental damages caused by a natural disaster or human activities, such as earthquakes, turbulent currents, anchoring and fishing gear [1,2]. In order to prevent such aggressive breaks to which the cables are exposed, they are often buried underground in shallow waters or placed in the seabed of deep waters. In this case, it must provide timely information on the present seabed environment, including the buried depth and sub-bottom profiles along the cable route in order to maintain the underwater cables.

For the underwater cable detection, dedicated devices, such as the underwater camera, side scan sonar, sub-bottom profiler and magnetic sensor, are often used. Compared to visual and acoustic methods working well for large diameter pipelines, sensing of electromagnetic fields is the most suitable method for locating the buried depth of a very thin cable and tracking the cable route regardless of whether the cable is buried underground or placed beneath the seabed [3,4]. In fact, magnetic sensing technology is applied to not only the detection of the underwater cables, but also bridge cables, steel cables, underground pipelines, etc. [5,6,7,8]. There are two types of methods adopted in practical applications of magnetic detection. The first is named the passive method, which relies on inducing alternating current (AC) onto a transmission line and detecting the parameters of the resulting electromagnetic field. This method requires onshore access to the line, as well as ensuring the continuity of the electric flow. The second is the active sensing method, which uses an active approach of emitting the magnetic field by onboard equipment to induce a current inside the ferromagnetic materials of the cables. After switching off the magnetic exciting source, the magnetic field produced by the remaining eddy current can be sensed. Although both methods require different hardware equipments, they have the similar detection mechanism in terms of sensing the magnetic field and need the carrier of hardware equipment [9].

Nevertheless, the location and inspection of the subsea telecommunication cables are quite difficult and dangerous. The diver can only remain in the water for a short period of time, which results in covering a lot less ground than the underwater vehicle, and it is difficult for a diver to operate in deep waters with limited visibility or steep sand-waves. In addition, the localization of the underwater cables based on a human’s judgment is susceptible to errors and not reliable in most cases [10]. Therefore, underwater vehicles equipped with magnetic sensors are often applied to detect the buried cables. On the one hand, when the remotely-operated vehicle (ROV) is deployed to track the underwater cables, mother vessels with high maneuver ability are required to follow and support the ROV persistently. Moreover, large onboard cable-handling devices, such as cable drums and winches, must be carried by the mother vessels. Therefore, the overall operating costs of ROVs are expensive, which hinders their applications. On the other hand, an autonomous underwater vehicle (AUV) without the support of mother vessels is becoming the most emerging vehicle in the field of marine inspection, such as offshore oil and gas exploration, underwater pipeline inspection, seafloor imaging and broad-area surveying of oceanic features [11,12,13,14,15,16,17,18,19]. As a typical application case, a series of AUV prototypes for detecting and tracking the underwater cables, such as AQUA EXPLORER 1000 (AE-1000) and AQUA EXPLORER 2 (AE-2), completed some sea trials in Hokkaido and the Taiwan Strait [2,20,21,22]. Although the AUV has no tether link and the offshore operating cost is reduced in the absence of a mother ship, it places greater demands on navigation accuracy and autonomy to control the vehicle cruising along the underwater cable.

In fact, the cable tracking control of an AUV in the horizontal plane can be achieved by changing the thrusts of the port and starboard thrusters or the angle of a pair of rudders. As described in [20], the cable tracking controller calculates the control inputs so that both the relative direction and the relative distance between the AUV and the cable become zero, and it essentially can be regarded as the classic path following problem, which requires the AUV to follow a desired geometric path with spatial convergence alone, but without any temporal specification [23,24,25,26,27]. There have been great efforts to develop the intelligent controllers for AUV path following in recent years. In [28], a nonlinear path following controller based on the backstepping technique for an under-actuated AUV is proposed; however, a singularity exists in the algorithm because the orthogonal projection of the vehicle onto the path is used to build the Serret–Frenet reference frame. This restriction can be relieved by introducing a virtual target moving along the path, which introduces an additional degree of freedom to eliminate the singularity [29,30,31]. Subsequently, the backstepping-based controller depending on an integrated control Lyapunov function is proposed in [26,32,33]. Similar backstepping and predictive techniques based on line-of-sight (LOS) guidance law are used in [34,35]. In [36], a sliding mode fuzzy controller is applied for the AUV-Hai-Min in shallow water in order to perform LOS guidance in the horizontal plane. The fuzzy controller and neural network controller for AUV tracking control are listed in [37,38,39,40,41,42] and the references therein. In addition, an adaptive integral line-of-sight (ILOS) guidance law is introduced to path following control when the vehicle is exposed to the unknown drift forces caused by waves, wind and ocean currents [43,44,45]. Similar methods are also applied in the path following control of unmanned surface vessels (USVs), and the readers can refer to the literature [46,47,48,49,50,51,52,53,54,55]. These intelligent controllers exhibit good performance in path following; however, they are not easy for extended application in the onboard controller due to the complicated inference and the sensitivity to the control parameters, especially appearing in the fuzzy controller and neural network controller. Considering that cable tracking and detection are practical and urgent tasks, it is necessary to adopt a simplified control method and to evaluate the effectiveness of the cable tracking controller through numerical simulations. To the best knowledge of the authors, there have been few studies reported on the comprehensive simulation regarding the underwater cable tracking.

**Figure 1:**
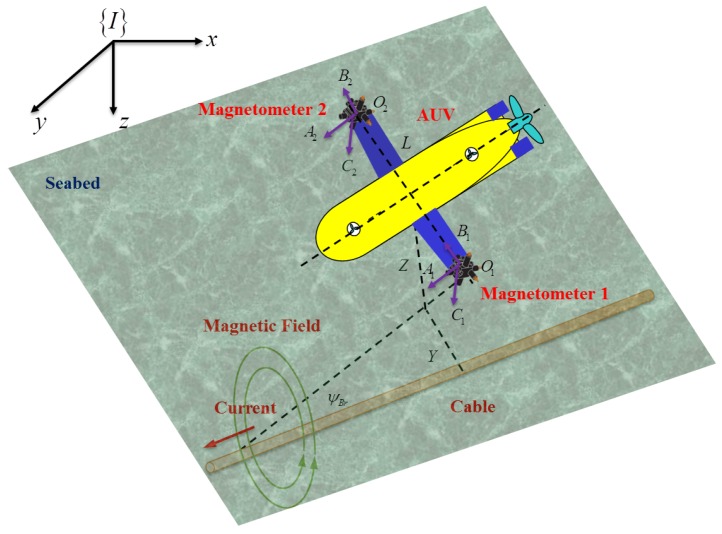
The yellow AUV equipped with two tri-axial magnetometers is tracking the orange cable. The induced electromotive forces in the *x*-axis, *y*-axis and *z*-axis of the tri-axial Magnetometer 1 and Magnetometer 2 are $\left(A_{1},B_{1},C_{1}\right)$ and $\left(A_{2},B_{2},C_{2}\right)$, respectively. The distance between those two magnetometers is $O_{1}O_{2}=L$, and {I} is the inertial frame. In addition, $\psi_{Br}$, *Y* and *Z* denote the heading deviation, horizontal offset and vertical distance between the AUV and the cable.

Motivated by the above considerations, this paper proposes a novel framework integrating the cable localization algorithm with the magnetic guidance and control algorithm to illustrate the automatic cable tracking performance of an inspection AUV without human beings in the loop. As shown in Figure 1, the relative position and orientation between the AUV and the cable are analyzed and calculated based on the sensing feedback of the double tri-axial magnetic arrays, such that the subsea cable is localized. Subsequently, a magnetic LOS guidance law is proposed based on the relative geometric relationship between the AUV and the cable, which is generated by cable localization information. Then, a simplified cable tracking controller using the feedback linearizing technique is designed to drive the AUV traveling along the cable based on the magnetic guidance law in order to enable the inspection of the buried cable environment. Finally, a comprehensive numerical simulation case is investigated to validate the effectiveness of the integrated magnetic localization, guidance and control algorithm on the envisioned underwater cable tracking and the potential protection of the seabed environment along the cable route.

The rest of the paper is organized as follows. The passive magnetic sensing principle and cable location method are presented in Section 2. In Section 3, the magnetic LOS guidance law is proposed for an under-actuated AUV, and then, the simplified cable tracking controller based on the feedback linearizing technique is designed. Numerical simulation results are given in Section 4 to illustrate the performance of the proposed AUV cable tracking system. Section 5 contains some concluding remarks and discusses some open problems that warrant further research.

## 2. Magnetic Sensing and Cable Localization

In this section, the passive method of the underwater cable measurements by two tri-axial magnetometers is introduced. Subsequently, the analytic deduction of cable localization including the heading deviation, buried depth and horizontal offset is presented based on the passive magnetic sensing principle, which is instrumental to design the magnetic LOS guidance law in the next section.

### 2.1. Passive Magnetic Sensing

When the AUV is required to track the underwater optical cables, low frequency (16–25 Hz) currents are first supplied to conductors in the cables, and then, the coaxial alternating magnetic field is produced by the currents. Subsequently, once the two tri-axial magnetometers placed in the wings of the AUV moving along the predefined path detect the magnetic field, the induced electromotive force of the closed coil in the tri-axial magnetometer will be generated and measured. According to the related theory of electromagnetic field, the induced electromotive force *ε* is the inverse ratio to the distance *R* between the close coil and the long straight cable [56], which can be expressed as:

(1) $$\varepsilon =\frac{k_{\varepsilon }}{R}$$

where $k_{\varepsilon }$ is constant and depends on the magnetic conductivity *μ*, the cross-sectional area *S* of the closed coil in the tri-axial magnetometer, the current changing rate $\dot{I}$ and the included angle *η* between the magnetic field lines and its normal to the cross-section.

### 2.2. Cable Location

**Figure 2:**
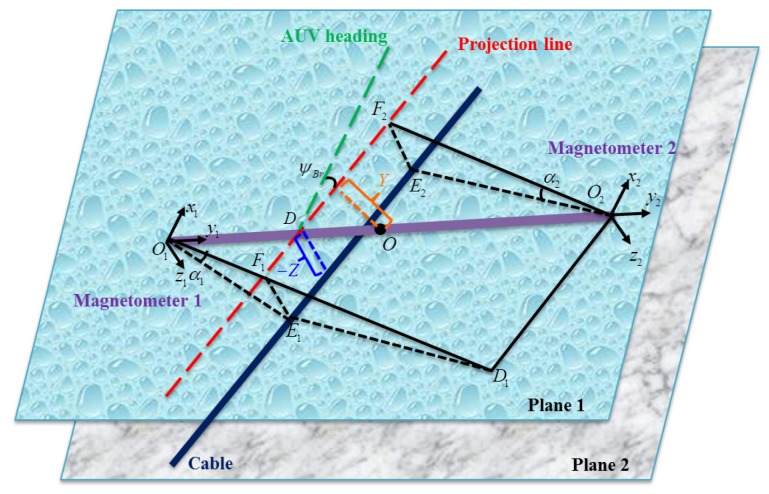
The yellow AUV equipped with two tri-axial magnetometers and the blue-black cable are located in Plane 1 and Plane 2, respectively. In Plane 1, the red dashed line denotes the projection line of the cable; the green dashed line denotes the AUV heading; and the purple line is the connection line between two tri-axial magnetometers, represented by $O_{1}x_{1}y_{1}z_{1}$ and $O_{2}x_{2}y_{2}z_{2}$, respectively. Note that their *x*-axes are parallel to the longitudinal axis directed from aft to fore of the AUV and the *z*-axes are directed from top to bottom. Constructing the auxiliary lines as $O_{1}E_{1}⊥E_{1}E_{2}$, $E_{1}F_{1}⊥O_{1}F_{1}$, $O_{2}E_{2}⊥E_{1}E_{2}$, $E_{2}F_{2}⊥O_{2}F_{2}$ and $O_{2}D_{1}⊥O_{1}D_{1}$ gives the following geometrical relationships for locating the cable: (1) $F_{1}E_{1}=F_{2}E_{2}=-Z$; (2) $▵O_{1}DF_{1}∼▵O_{2}DF_{2}$; (3) $F_{1}D_{1}=O_{2}F_{2}$; (4) $∠O_{2}O_{1}D_{1}=\psi_{Br}$.

In this paper, we assume that the cable is located between the two tri-axial magnetometers, as shown in Figure 2. Based on this assumption, we clearly show how to locate the cables using an analytic method. The same result can be obtained if the cable is located on the left-hand side or on the right-hand side of the two tri-axial magnetometers.

#### 2.2.1. Heading Deviation

**Figure 3:**
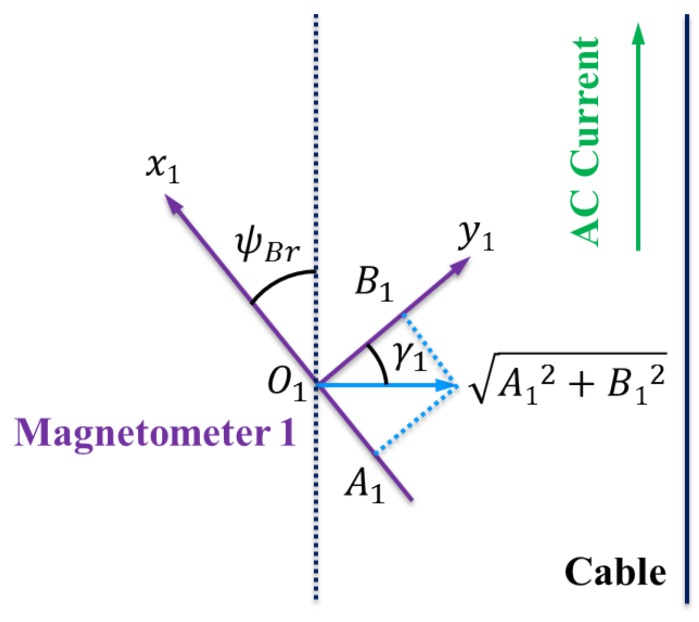
For the tri-axial Magnetometer 1, the induced electromotive force projected in the horizontal plane is perpendicular to the cable. Obviously, $\tan\gamma_{1}=\frac{A_{1}}{B_{1}}$ and $\gamma_{1}=\psi_{Br}$.

The induced electromotive force projected in the horizontal plane is depicted in Figure 3. Note that if the AUV heading is parallel to the cable, the induced electromotive force component in the *x*-axis of the tri-axial magnetometer is zero. If the AUV heading is perpendicular to the cable, the induced electromotive force component in the *y*-axis of the tri-axial magnetometer is zero. In general, the heading deviation between the direction of the cable and the heading of the AUV can be represented by the angle $\psi_{Br}$ as follows:

(2) $$\psi_{Br}=\arctan\left(\frac{A_{1}}{B_{1}}\right)$$

#### 2.2.2. Buried Depth

**Figure 4:**
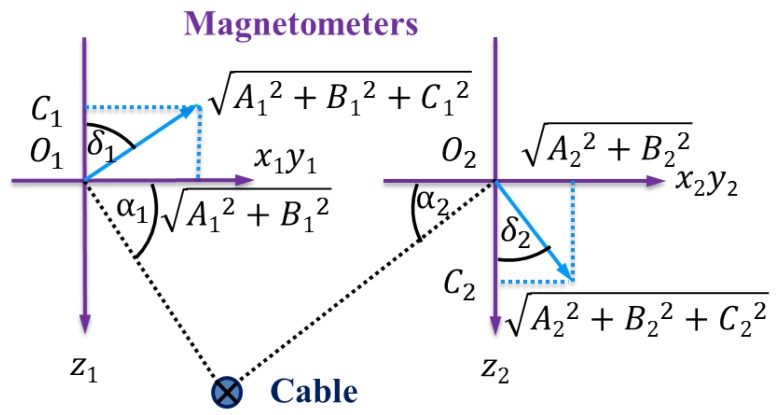
Assume that the cable is located in the middle of two tri-axial magnetometers; there is $C_{1}<0$ and $C_{2}>0$. Hence, $\tan\left(\delta_{1}\right)=\frac{\sqrt{A_{1}^{2}+B_{1}^{2}}}{-C_{1}}$ and $\tan\left(\delta_{2}\right)=\frac{\sqrt{A_{2}^{2}+B_{2}^{2}}}{C_{2}}$. In addition, $\alpha_{1}=\delta_{1}$ and $\alpha_{2}=\delta_{2}$.

From an elevation view in the direction of the input current shown in Figure 4, there is:

(3) $$\left\{\begin{array}{c} \tan\left(\alpha_{1}\right)=\frac{\sqrt{A_{1}^{2}+B_{1}^{2}}}{-C_{1}}\\ \tan\left(\alpha_{2}\right)=\frac{\sqrt{A_{2}^{2}+B_{2}^{2}}}{C_{2}} \end{array}\right.$$

Considering the triangles $▵O_{1}F_{1}E_{1}$ and $▵O_{2}F_{2}E_{2}$, it has:

(4) $$\left\{\begin{array}{c} O_{1}F_{1}=\frac{F_{1}E_{1}}{\tan(\alpha_{1})}\\ O_{2}F_{2}=\frac{F_{2}E_{2}}{\tan(\alpha_{2})} \end{array}\right.$$

In the horizontal Plane 1 of Figure 2, the point $F_{1}$ divides the segment $O_{1}D_{1}$ into two parts. Hence,

(5) $$O_{1}F_{1}+F_{1}D_{1}=O_{1}D_{1}$$

In view of Equation (4), $F_{1}E_{1}=F_{2}E_{2}=-Z$ and $F_{1}D_{1}=O_{2}F_{2}$, Equation (5) can be rewritten as:

(6) $$\frac{-Z}{\tan\alpha_{1}}+\frac{-Z}{\tan\alpha_{2}}=L\cos\left(\psi_{Br}\right)$$

where *Z* denotes the vertical distance between the AUV and the cable and *L* denotes the distance between two tri-axial magnetometers shown in Figure 1.

Therefore, using the magnetic sensing feedback data, the vertical distance *Z* can be continuously calculated by the onboard controller online as:

(7) $$Z=\frac{B_{1}\sqrt{A_{2}^{2}+B_{2}^{2}}}{C_{1}\sqrt{A_{2}^{2}+B_{2}^{2}}-C_{2}\sqrt{A_{1}^{2}+B_{1}^{2}}}L$$

Further, the buried depth *d* of the underwater cable is:

(8) d=Z+h

where *h* denotes the vertical distance between the AUV and the seabed and can be easily measured by the altimeter installed in the AUV. Furthermore, the AUV equipped with the sub-bottom profiler can inspect and record the sub-bottom information along the cable route, which provides the complete and timely information to improve and protect the seabed environment of the cable by taking counterpart measures, such as setting prohibited fishing/anchoring zones based on the cable route information inspected by the AUV and increasing the buried depth by filling the gap in the shallow or uncovered cable route.

#### 2.2.3. Horizontal Offset

In Figure 2, $▵O_{1}DF_{1}∼▵O_{2}DF_{2}$ renders

(9) $$\frac{O_{1}D}{O_{2}D}=\frac{O_{1}F_{1}}{O_{2}F_{2}}$$

Substituting Equation (4) into Equation (9) leads to:

(10) $$\frac{O_{1}D}{O_{2}D}=\frac{\tan(\alpha_{2})}{\tan(\alpha_{1})}$$

Consider the following geometrical relationships:

(11) $$\left\{\begin{array}{c} O_{1}D=O_{1}O-OD\\ O_{2}D=O_{2}O+OD\\ O_{1}O=O_{2}O \end{array}\right.$$

This yields that:

(12) $$OD=\frac{C_{1}\sqrt{A_{2}^{2}+B_{2}^{2}}+C_{2}\sqrt{A_{1}^{2}+B_{1}^{2}}}{-C_{1}\sqrt{A_{2}^{2}+B_{2}^{2}}+C_{2}\sqrt{A_{1}^{2}+B_{1}^{2}}}\frac{L}{2}$$

Consequently, the horizontal offset *Y* between the AUV and the cable can be calculated as:

(13) $$Y=\frac{C_{1}\sqrt{A_{2}^{2}+B_{2}^{2}}+C_{2}\sqrt{A_{1}^{2}+B_{1}^{2}}}{-C_{1}\sqrt{A_{2}^{2}+B_{2}^{2}}+C_{2}\sqrt{A_{1}^{2}+B_{1}^{2}}}\frac{L\cos(\psi_{Br})}{2}$$

According to the heading deviation and horizontal offset between the AUV and cable, the AUV can autonomously steer itself with the onboard controller to move towards the subsea cable and inspect the seabed environment along the buried cable route. Note that the same location results including Equations (2), (8) and (13) can be obtained whether the cable is located on the left-hand side or on the right-hand side of two tri-axial magnetometers, which means that the cable location method in this paper does not place a requirement on the relative position between two tri-axial magnetometers and the cable. Therefore, the AUV will not need to switch the algorithm, and there is no transition in the coincident solving algorithm that prevents from a potential unstable behavior.

## 3. Magnetic Guidance and Tracking Control

In this section, the modeling of an under-actuated AUV is briefly introduced. Based on the magnetic sensing and localization, the dedicated magnetic LOS guidance law is proposed for the cable tracking control of the under-actuated vehicle, and then, the simplified dynamic controller relying on the feedback linearizing technique is designed to drive the surge speed and yaw angle of the AUV to attain the desired profiles, such that the under-actuated AUV tracks and detects along the underwater cable.

### 3.1. AUV Modeling

Suppose the AUV tracking the underwater cable is equipped with a stern propeller and two pair of rudders. Therefore, the AUV is under-actuated in sway, heave and roll, since there are no thrusters to directly contribute the corresponding force and moments [57]. In addition, the roll and pitch motions of the vehicle are ignored due to a large metacentric height and relatively large contact area of two wide wings in the water. During the cable tracking mission, the vehicle firstly dives to the suitable depth relative to the underwater cable, such that it is able to catch the magnetic signals. In this sense, we focus on how to control the AUV in the fixed-depth plane to track the cable under the magnetic guidance.

Following the standard notations, the general kinematic and dynamic model of the AUV in the horizontal plane can be described by the motion components in surge, sway and yaw directions [58]. Hence, the kinematic equations take the form:

(14) $$\left\{\begin{array}{c} \dot{x}=u\cos\left(\psi_{B}\right)-v\sin\left(\psi_{B}\right)\\ \dot{y}=u\sin\left(\psi_{B}\right)+v\cos\left(\psi_{B}\right)\\ \dot{\psi_{B}}=r \end{array}\right.$$

where *x* and *y* are the coordinates of its center of mass expressed in the inertial frame {I} and $\psi_{B}$ defines its orientation (yaw angle). Surge speed *u*, sway speed *v* and yaw speed *r* denote the AUV body-fixed linear and angular velocities with respect to the inertial frame.

Neglecting the motions in heave, roll and pitch, the three-degrees-of-freedom (3-DOF) dynamic equations of the AUV in the horizontal plane are simplified as:

(15) $$\left\{\begin{array}{c} \dot{u}=\frac{m_{22}}{m_{11}}vr-\frac{d_{11}}{m_{11}}u-\sum_{i=2}^{3}\frac{d_{ui}}{m_{11}}{\left|u\right|}^{i-1}u+\frac{\tau_{u}}{m_{11}}\\ \dot{v}=-\frac{m_{11}}{m_{22}}ur-\frac{d_{22}}{m_{22}}v-\sum_{i=2}^{3}\frac{d_{vi}}{m_{22}}{\left|v\right|}^{i-1}v\\ \dot{r}=\frac{m_{11}-m_{22}}{m_{33}}uv-\frac{d_{33}}{m_{33}}r-\sum_{i=2}^{3}\frac{d_{ri}}{m_{33}}{\left|r\right|}^{i-1}r+\frac{\tau_{r}}{m_{33}} \end{array}\right.$$

where $m_{\left(\cdot \right)}$ expresses the hydrodynamic derivatives of the system and $d_{\left(\cdot \right)}$ captures the hydrodynamic damping effects. $\tau_{u}$ denotes the external force acting on the AUV in the surge direction, and $\tau_{r}$ denotes the external torque about the *z*-axis of the AUV. Note that the AUV is under-actuated since there is no control contribution in the sway direction.

### 3.2. Guidance and Control Design

**Figure 5:**
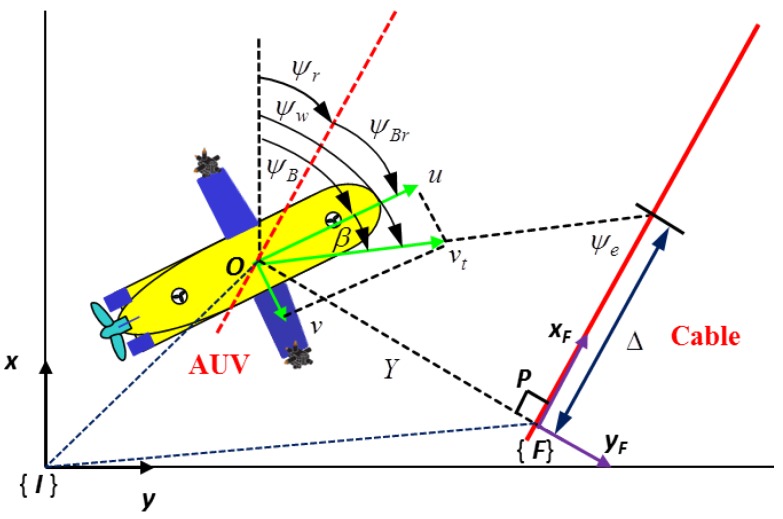
Let the position and course angle of the under-actuated AUV be denoted by $O={\left(x,y,\psi_{w}\right)}^{⊤}$ in the inertial frame {I}, and let the position and orientation of the projection point of the AUV on the path be denoted by $P={\left(x_{r},y_{r},\psi_{r}\right)}^{⊤}$ in the inertial frame {I}. The side-slip angle $\beta =\arctan(\frac{v}{u})$. In the Serret–Frenet frame {F} with the origin at *P*, the LOS guidance angle is defined as $\arctan(\frac{-Y}{\Delta })$, where *Y* is the horizontal offset of the AUV relative to the cable, and the look ahead distance Δ is constant. The choice of Δ is instrumental to shape the AUV moving towards the straight-line path.

The problem of cable tracking control for an under-actuated AUV can be formulated as follows: for a straight long underwater cable shown in Figure 5, develop feedback control laws for the available force and moment acting on an under-actuated AUV, such that the horizontal offset *Y* between the AUV and the cable reduces to zero and the resultant velocity vector of the AUV is aligned with the tangent vector of the cable, namely:

(16) $$\left\{\begin{array}{c} \lim_{t\to \infty }Y=0\\ \lim_{t\to \infty }\psi_{e}=0 \end{array}\right.$$

with $\psi_{e}=\psi_{w}-\psi_{r}$, which is taken as the course tracking error between the course angle $\psi_{w}$ of the AUV and the direction angle $\psi_{r}$ of the cable.

#### 3.2.1. Magnetic Guidance

When being aware of the heading deviation between the direction of the cable and the heading of the AUV, the direction of the cable can be continuously calculated by the AUV since its heading angle can be easily measured. Thus, the center of the AUV can be projected on the cable route as shown in Figure 5. Let the virtual projection point *P* denoted by ${\left(x_{r},y_{r},\psi_{r}\right)}^{⊤}$ in {I} be the origin of the Serret–Frenet frame {F}. The *x*-axis of the frame {F} is along the direction of the cable, and the *y*-axis is the normal of the cable route. Therefore, the cable tracking error vector $\varepsilon ={\left(0,Y,\psi_{e}\right)}^{⊤}$ built in the frame {F} can be written as:

(17) $$\begin{array}{c} \left[\begin{array}{c} 0\\ Y\\ \psi_{e} \end{array}\right]=\left[\begin{array}{ccc} \cos(\psi_{r}) & \sin(\psi_{r}) & 0\\ -\sin(\psi_{r}) & \cos(\psi_{r}) & 0\\ 0 & 0 & 1 \end{array}\right]\left[\begin{array}{c} x-x_{r}\\ y-y_{r}\\ \psi_{w}-\psi_{r} \end{array}\right] \end{array}$$

Differentiating the error vector in Equation (17), it yields the error dynamics:

(18) $$\begin{array}{c} \left[\begin{array}{c} 0\\ \dot{Y}\\ {\dot{\psi }}_{e} \end{array}\right]=-{\dot{\psi }}_{r}\left[\begin{array}{ccc} \sin(\psi_{r}) & -\cos(\psi_{r}) & 0\\ \cos(\psi_{r}) & \sin(\psi_{r}) & 0\\ 0 & 0 & 0 \end{array}\right]\left[\begin{array}{c} x-x_{r}\\ y-y_{r}\\ \psi_{w}-\psi_{r} \end{array}\right]+\left[\begin{array}{ccc} \cos(\psi_{r}) & \sin(\psi_{r}) & 0\\ -\sin(\psi_{r}) & \cos(\psi_{r}) & 0\\ 0 & 0 & 1 \end{array}\right]\left[\begin{array}{c} \dot{x}-{\dot{x}}_{r}\\ \dot{y}-{\dot{y}}_{r}\\ {\dot{\psi }}_{w}-{\dot{\psi }}_{r} \end{array}\right] \end{array}$$

Assume the virtual projection point *P* has the same kinematic property of the AUV, which means that $P={\left(x_{r},y_{r},\psi_{r}\right)}^{⊤}$ fulfills the kinematic constraints in Equation (14). After tedious computations, the error dynamics in Equation (18) can be rewritten as follows:

(19) $$\begin{array}{c} \left[\begin{array}{c} 0\\ \dot{Y}\\ {\dot{\psi }}_{e} \end{array}\right]=\left[\begin{array}{c} -v_{r}+v_{t}\cos\left(\psi_{e}\right)\\ v_{t}\sin\left(\psi_{e}\right)\\ {\dot{\psi }}_{w}-{\dot{\psi }}_{r} \end{array}\right] \end{array}$$

where $v_{t}=\sqrt{u^{2}+v^{2}}>0$ is the resultant velocity of the AUV and $v_{r}$ is the virtual velocity of the point *P*.

As one of the main objectives of the cable tracking control is to drive the horizontal offset *Y* to zero, the following candidate Lyapunov function can be considered:

(20) $$V=\frac{1}{2}Y^{2}$$

Resorting to the error dynamics model in Equation (19), the derivative of *V* is:

(21) $$\dot{V}=v_{t}Y\sin\left(\psi_{e}\right)$$

According to the LOS guidance principle [33,58,59], if the course tracking error angle $\psi_{e}$ is driven to be equal to the LOS guidance angle as illustrated in Figure 5, the AUV will move towards and then follow the cable with imitated helmsman behaviors under the LOS guidance. Through this intuitive design, we can straightforwardly re-define the course tracking error as:

(22) $$\psi_{e}=\arctan\left(\frac{-Y}{\Delta }\right)$$

where the look ahead distance Δ>0, and it is usually specified as two vehicle’s length. The re-defined course tracking error $\psi_{e}$ will be used to construct the desired course/yaw angle of the AUV for the cable tracking later.

Substituting Equation (22) into Equation (21) leads to:

(23) $$\dot{V}=-\frac{v_{t}Y^{2}}{\sqrt{Y^{2}+\Delta^{2}}}$$

Therefore, $\dot{V}<0$ anywhere except the origin. From the Lyapunov stability theorem, it is concluded that:

(24) $$\lim_{t\to \infty }Y=0$$

Moreover, by revisiting Equation (22), $\lim_{t\to \infty }Y=0$ renders that:

(25) $$\lim_{t\to \infty }\psi_{e}=0$$

As $\psi_{e}=\psi_{w}-\psi_{r}$ and $\psi_{B}=\psi_{w}-\beta$, by considering the course tracking error in Equation (22) under the LOS guidance, the desired yaw angle $\psi_{Bd}$ can be designed as:

(26) $$\psi_{Bd}=\arctan\left(\frac{-Y}{\Delta }\right)+\psi_{r}-\beta$$

where $\beta =\arctan(\frac{v}{u})$ is the side-slip angle of the AUV.

Note that as the parameterized equation of the desired tracking path of the cable is unknown in advance, the course angle $\psi_{r}$ of the cable path cannot be solved by the trigonometric function $\arctan(\frac{{\dot{y}}_{r}}{{\dot{x}}_{r}})$ as proposed in the classic path following control design in [23,28,30,33]. Yet, it can be obtained indirectly through the magnetic sensing as follows:

(27) $$\psi_{r}=\psi_{B}-\psi_{Br}$$

As $\psi_{Br}$ is determined by the electromagnetic signals in Equation (2), the magnetic LOS guidance law represented by the desired yaw angle $\psi_{Bd}$ in Equation (26) can be rewritten as:

(28) $$\psi_{Bd}=\arctan\left(\frac{-Y}{\Delta }\right)-\arctan\left(\frac{A_{1}}{B_{1}}\right)+\psi_{B}-\beta$$

Since the cable tracking error vector is reduced to 0 from Equation (24) and Equation (25), it is concluded that the AUV is able to track the underwater cable well under the magnetic LOS guidance law in Equation (28).

#### 3.2.2. Cable Tracking Control

As the under-actuated AUV investigated in this paper is equipped with a stern propeller and two pair of rudders, but without any lateral thrusters, the surge speed *u* can be driven to the desired speed $u_{d}$, while the side-slip angle cannot rigorously converge to a desired angle. Considering $u=v_{t}\cos\left(\beta \right)$, the desired surge speed $u_{d}$ can be designed as:

(29) $$u_{d}=v_{td}\cos\left(\beta \right)$$

where $v_{td}$ is the desired resultant velocity of the AUV. Obviously, when $u\to u_{d}$, $v_{t}\to v_{td}$ owing to $u=v_{t}\cos\left(\beta \right)$ and Equation (29).

Resorting to the feedback linearizing technique to eliminate the system nonlinearity in case the model parameters of the AUV are known in advance, we can design the following control law:

(30) $$\left\{\begin{array}{c} \tau_{u}=m_{11}\left(-\frac{m_{22}}{m_{11}}vr+\frac{d_{11}}{m_{11}}u+\sum_{i=2}^{3}\frac{d_{ui}}{m_{11}}{\left|u\right|}^{i-1}u+{\dot{u}}_{d}+k_{Pu}e_{u}\right)\\ \tau_{r}=m_{33}\left(-\frac{m_{11}-m_{22}}{m_{33}}uv+\frac{d_{33}}{m_{33}}r+\sum_{i=2}^{3}\frac{d_{ri}}{m_{33}}{\left|r\right|}^{i-1}r+{\ddot{\psi }}_{Bd}+k_{D\psi }{\dot{e}}_{\psi }+k_{P\psi }e_{\psi }\right) \end{array}\right.$$

with:

(31) $$\left\{\begin{array}{l} e_{u}=u_{d}-u\\ e_{\psi }=\psi_{Bd}-\psi_{B} \end{array}\right.$$

Clearly, the following derivative of the side-slip angle $\dot{\beta }$ is requested for the control computation of the under-actuated AUV in Equation (30):

(32) $$\dot{\beta }=\frac{u\dot{v}-\dot{u}v}{v_{t}^{2}}$$

Assume that the long cable tracked by the AUV is straight, which means that $\psi_{r}=-\arctan\left(\frac{A_{1}}{B_{1}}\right)+\psi_{B}$ is constant and its derivative is zero. Normally, the desired resultant velocity of the under-actuated AUV is set to be constant. Hence, the derivatives of the desired surge velocity ${\dot{u}}_{d}$ and yaw angle error ${\dot{e}}_{\psi }$ can be computed as:

(33) $$\left\{\begin{array}{c} {\dot{u}}_{d}=-v_{td}\sin\left(\beta \right)\frac{u\dot{v}-\dot{u}v}{v_{t}^{2}}\\ {\dot{e}}_{\psi }=-\frac{v_{t}\Delta \sin\left(\psi_{Br}+\beta \right)}{Y^{2}+\Delta^{2}}-\frac{u\dot{v}-\dot{u}v}{v_{t}^{2}}-r \end{array}\right.$$

where $\dot{u}$ and $\dot{v}$ are defined in Equation (15). In addition, the second derivative of the desired yaw angle ${\ddot{\psi }}_{Bd}$ is estimated by backward Euler method:

(34) $${\ddot{\psi }}_{Bd}\left(n\right)=\frac{{\dot{\psi }}_{Bd}\left(n\right)-{\dot{\psi }}_{Bd}\left(n-1\right)}{dt}$$

where ${\dot{\psi }}_{Bd}\left(n\right)={\dot{e}}_{\psi }\left(n\right)+r\left(n\right)$, *n* and d*t* are the sampling index and the control period of the closed loop system, respectively.

Substituting Equation (30) into Equation (15) leads to:

(35) $$\left\{\begin{array}{c} {\dot{e}}_{u}+k_{Pu}e_{u}=0\\ {\ddot{e}}_{\psi }+k_{D\psi }{\dot{e}}_{\psi }+k_{P\psi }e_{\psi }=0 \end{array}\right.$$

Further, the corresponding characteristic equations of the whole closed loop control system are described as:

(36) $$\left\{\begin{array}{c} s+k_{Pu}=0\\ s^{2}+k_{D\psi }s+k_{P\psi }=0 \end{array}\right.$$

According to the Routh–Hurwitz criterion, all of the roots of the characteristic equations in Equation (36) will be located in the left half region of the complex plane if $k_{Pu}>0$, $k_{P\psi }>0$ and $k_{D\psi }>0$. Therefore, let the control laws be given by Equation (30), $e_{u}\left(t\right)\to 0$ and $e_{\psi }\left(t\right)\to 0$ when t→∞, namely the surge speed and yaw angle of the AUV will converge asymptotically to their desired profiles.

## 4. Numerical Simulation

In order to illustrate the cable tracking performance of the proposed magnetic guidance and tracking control framework, as well as the feedback linearizing controller in this paper, the surrounding magnetic field of the under-actuated AUV is first simulated in this section, and then, numerical cases of tracking a straight cable buried seabed by the AUV are investigated with the simulation results given in detail.

### 4.1. Magnetic Field Simulation

Suppose that the straight cable tracked by the under-actuated AUV is parameterized by:

(37) $$\left\{\begin{array}{c} x_{r}=\varpi \\ y_{r}=k\varpi +b\\ z_{r}=c \end{array}\right.$$

with ϖ(0)=0[m], c=150[m], k=0.5 and b=1.

**Figure 6:**
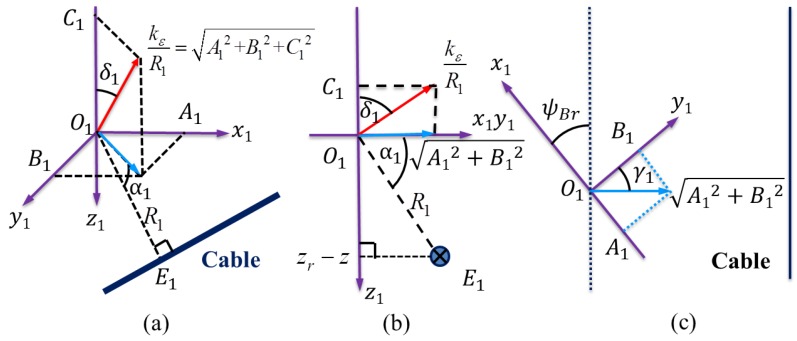
(**a**) Three-dimensional view of the magnetic sensing; (**b**) Two-dimensional vertical view; and (**c**) Two-dimensional horizontal view. There are $\sqrt{A_{1}^{2}+B_{1}^{2}}=\frac{k_{\varepsilon }}{R_{1}}\sin\left(\delta_{1}\right)$ with $\delta_{1}=\alpha_{1}=\arcsin\left(\frac{z_{r}-z}{R_{1}}\right)$, $A_{1}=\sqrt{A_{1}^{2}+B_{1}^{2}}\sin\left(\gamma_{1}\right)$ with $\gamma_{1}=\psi_{Br}=\psi_{B}-\psi_{r}$, $B_{1}=\sqrt{A_{1}^{2}+B_{1}^{2}}\cos\left(\gamma_{1}\right)$, and $C_{1}=\sqrt{\frac{k_{\varepsilon }^{2}}{R_{1}^{2}}-A_{1}^{2}-B_{1}^{2}}=\frac{k_{\varepsilon }}{R_{1}}\sqrt{\left(1-\frac{{\left(z_{r}-z\right)}^{2}}{R_{1}^{2}}\right)}$.

If the position of the AUV is (x,y,z), the induced electromotive forces measured by the port tri-axial Magnetometer 1 coordinated at $\left(x+\frac{L}{2}\sin\left(\psi_{B}\right),y-\frac{L}{2}\cos\left(\psi_{B}\right),z\right)$ as shown in Figure 6 are:

(38) $$\begin{array}{c} \left[\begin{array}{c} A_{1}\\ B_{1}\\ C_{1} \end{array}\right]=\left[\begin{array}{c} \frac{k_{\varepsilon }}{R_{1}}\frac{z_{r}-z}{R_{1}}\sin\left(\psi_{B}-\arctan\left(k\right)\right)\\ \frac{k_{\varepsilon }}{R_{1}}\frac{z_{r}-z}{R_{1}}\cos\left(\psi_{B}-\arctan\left(k\right)\right)\\ \frac{k_{\varepsilon }}{R_{1}}\frac{-k\left(x+\frac{L}{2}\sin\left(\psi_{B}\right)\right)+\left(y-\frac{L}{2}\cos\left(\psi_{B}\right)\right)-b}{\sqrt{k^{2}+1}R_{1}} \end{array}\right]+f_{noi1} \end{array}$$

where $R_{1}=\sqrt{\frac{(k{\left(x+\frac{L}{2}\sin\left(\psi_{B}\right)-\left(y-\frac{L}{2}\cos\left(\psi_{B}\right)\right)+b\right)}^{2}}{k^{2}+1}+{\left(z_{r}-z\right)}^{2}}$ represents the distance between Magnetometer 1 and the cable and $f_{noi1}$ denotes the sensor noise on Magnetometer 1. The forces measured by the starboard tri-axial Magnetometer 2 coordinated at $\left(x-\frac{L}{2}\sin\left(\psi_{B}\right),y+\frac{L}{2}\cos\left(\psi_{B}\right),z\right)$ are:

(39) $$\begin{array}{c} \left[\begin{array}{c} A_{2}\\ B_{2}\\ C_{2} \end{array}\right]=\left[\begin{array}{c} \frac{k_{\varepsilon }}{R_{2}}\frac{z_{r}-z}{R_{2}}\sin\left(\psi_{B}-\arctan\left(k\right)\right)\\ \frac{k_{\varepsilon }}{R_{2}}\frac{z_{r}-z}{R_{2}}\cos\left(\psi_{B}-\arctan\left(k\right)\right)\\ \frac{k_{\varepsilon }}{R_{2}}\frac{-k\left(x-\frac{L}{2}\sin\left(\psi_{B}\right)\right)+\left(y+\frac{L}{2}\cos\left(\psi_{B}\right)\right)-b}{\sqrt{k^{2}+1}R_{2}} \end{array}\right]+f_{noi2} \end{array}$$

where $R_{2}=\sqrt{\frac{(k{\left(x-\frac{L}{2}\sin\left(\psi_{B}\right)-\left(y+\frac{L}{2}\cos\left(\psi_{B}\right)\right)+b\right)}^{2}}{k^{2}+1}+{\left(z_{r}-z\right)}^{2}}$ represents the distance between Magnetometer 2 and the cable and $f_{noi2}$ denotes the sensor noise on Magnetometer 2.

By using the formulas for the induced electromotive forces in Equations (38) and (39), the heading deviation in Equation (2), the buried depth in Equation (8) and the horizontal offset in Equation (13) of the underwater cable can be computed in the simulated situation of magnetic sensing.

### 4.2. Cable Tracking Results

The hydrodynamic parameters of the under-actuated AUV are listed in Table 1. When executing the cable tracking task, the AUV is first required to dive and then cruise in the fixed-depth plane of 145 m. Assume that its position and posture is ${\left[x\left(0\right),y\left(0\right),\psi \left(0\right)\right]}^{⊤}$ = ${\left[0\;m,-20\;m,\frac{\pi }{4}\;\text{rad}\right]}^{⊤}$ when the AUV detects the magnetic field, and the corresponding velocity is ${\left[u\left(0\right),v\left(0\right),r\left(0\right)\right]}^{⊤}$ = ${\left[1.2\;m/s,0\;m/s,0\;\text{rad}/s\right]}^{⊤}$.

**Figure 7:**
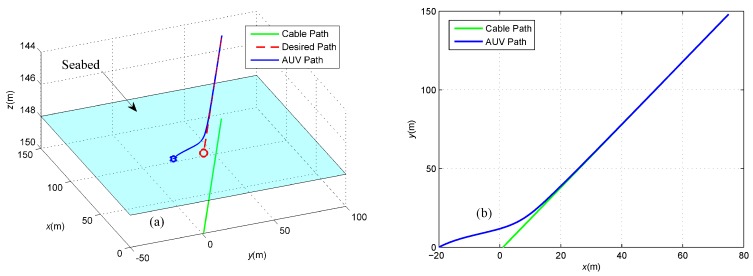
(**a**) Three-dimensional view of the cable tracking; and (**b**) Two-dimensional projection in the *X*-*Y*plane. These two pictures intuitively describe the cable tracking process, where the green line represents the underwater cable, the red dashed line represents the desired tracking path and the blue line represents the actual path of the under-actuated AUV.

**Figure 8:**
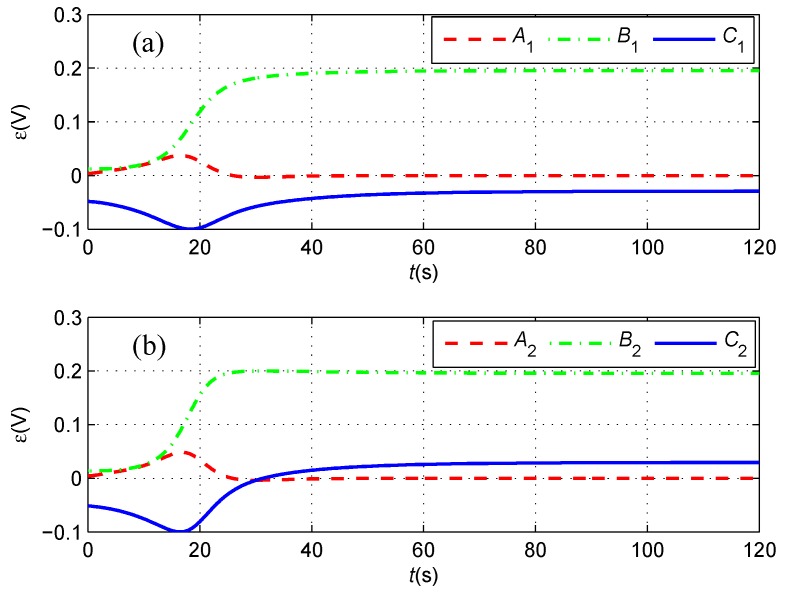
(**a**) The induced electromotive forces measured by the port tri-axial magnetometer; and (**b**) The induced electromotive forces measured by the starboard tri-axial magnetometer in the case of $k_{\varepsilon }=1$. The red dashed line, the green dash dotted line and the blue line represent the force components along the *x*-axis, *y*-axis and *z*-axis of the tri-axial magnetometer, respectively.

First, we assume that there is no sensor noise on the magnetometers, namely $f_{noi1}=f_{noi2}=0$. In this case, the cable tracking path is described in Figure 7. Obviously, the under-actuated AUV starting from the initial position marked by a blue pentagram in the fixed-depth plane moves towards, converges to and finally follows the desired red path. The transitions of the induced electromotive forces in the tracking period are described in Figure 8, where both the force components along the *x*-axis converge to zero and the components along the *y*-axis converge to the same constant. This means that the yaw angle of the AUV is identical to the buried orientation of the cable according to Equation (2). In addition, the components along the *z*-axis have the same size, but different directions at the final stages of tracking the cable, which indicates that the cable is located below in the middle of two tri-axial magnetometers installed in the AUV.

**Figure 9:**
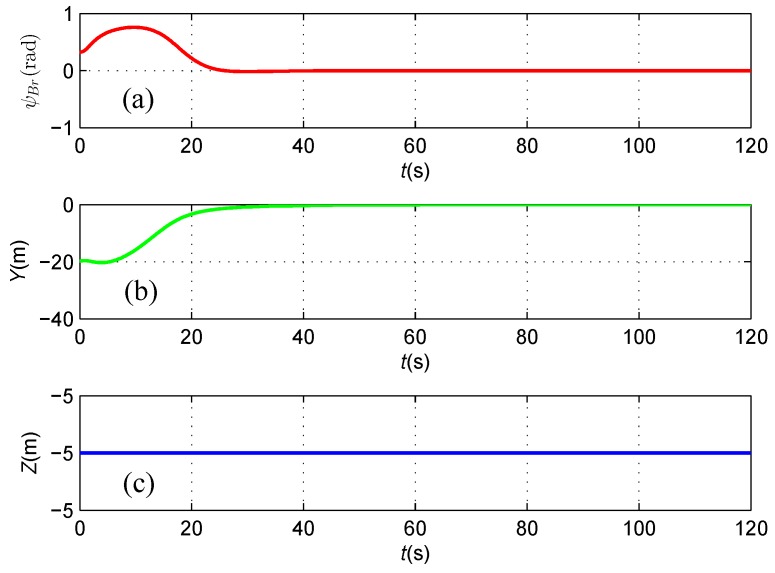
(**a**) The angle $\psi_{Br}$ between the AUV heading and the direction of the cable; (**b**) The horizontal offset *Y* between the AUV and the cable; and (**c**) The vertical distance *Z* between the AUV and the cable.

As shown in Figure 9, both the heading deviation between the direction of the cable and the AUV heading and the horizontal offset asymptotically reduce to zero, which illustrates that the AUV follows the cable route with its heading aligned with the orientation of the straight cable under the proposed feedback linearizing controller based on the magnetic sensor guidance. Consequently, the mission of inspecting and protecting the seabed environment along the cable route could be achieved by using the under-actuated AUV carried with two tri-axial magnetometers.

**Figure 10:**
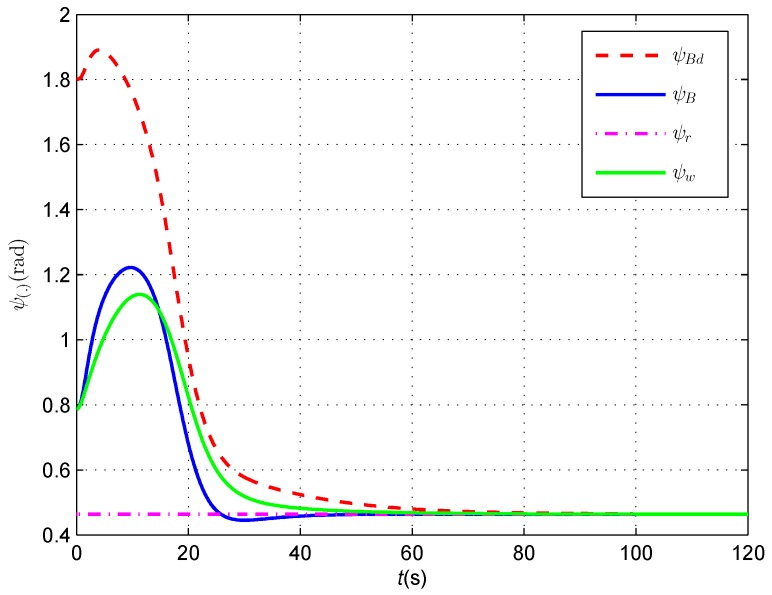
The red dashed line, the blue line and the green line represent the desired yaw angle, the actual yaw angle and the course angle of the AUV, respectively, and the pink dash dotted line represents the orientation angle of the cable.

In the whole tracking process, the yaw angle and course angle of the AUV are shown in Figure 10. Note that the desired yaw angle of the AUV keeps changing under the magnetic guidance law in spite of tracking the straight cable with the constant orientation. Besides, the yaw angle and course angle are not identical in the first 60 s, which indicates that the under-actuated AUV continuously changes its heading under the yaw moment and is subjected to the side-slip effects.

**Figure 11:**
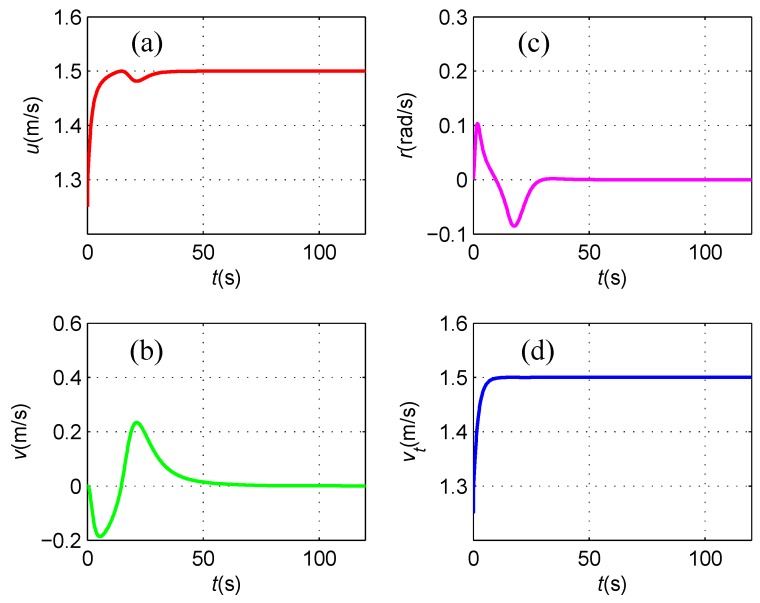
(**a**) Surge linear velocity *u*; (**b**) Sway linear velocity *v*; (**c**) Yaw angular velocity *r*; and (**d**) Resultant velocity $v_{t}$.

The linear and angular velocities are depicted in Figure 11, where the under-actuated AUV reaches the desired resultant velocity of 1.5 m/s. As mentioned before, the side-slip effects lead to a nonzero sway velocity *v* in the first 60 s.

**Figure 12:**
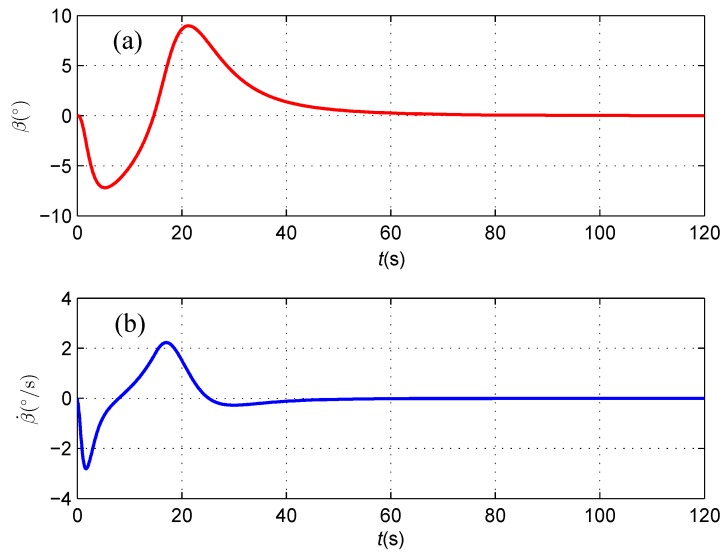
(**a**) Side-slip angle *β*; and (**b**) The derivative of the side-slip angle $\dot{\beta }$.

Although the side-slip angle finally converges to zero while the AUV follows the straight-line cable, Figure 12 clearly shows that the side-slip effect of the under-actuated AUV cannot be ignored during the cable tracking stage, as the maximum value of *β* is 8.9°, and its derivative also varies, which is up to 2.2°/s. It is concluded that the computation on the side-slip angle of the under-actuated AUV considered in this research is valuable.

**Figure 13:**
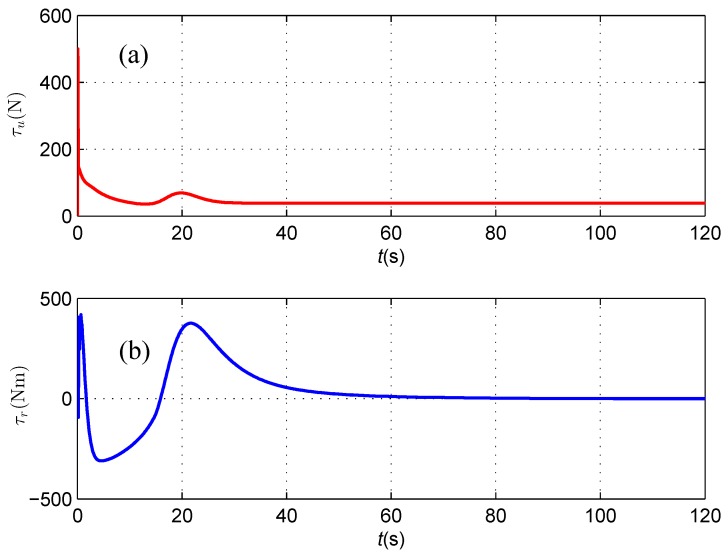
(**a**) Surge control force $\tau_{u}$; and (**b**) Yaw control moment $\tau_{r}$.

The available control inputs of the AUV including the surge force and yaw moment are depicted in Figure 13, where they keep changing against the position and orientation errors of the subsea cable tracking.

Second, for numerically validating the robustness of the proposed guidance and control algorithm in the presence of sensor noise, we assume that the simulation sensor models show periodic variation after the assumed data filtering procedure to the raw sensor data. It is well known that any complicated periodic function can be expanded in a series of simple *sine* and *cosine* functions by Fourier transformation. Hence, we choose suitable noise models $f_{noi1},f_{noi2}$ as follows:

(40) $$\begin{array}{cc} f_{noi1} & =f_{noi2}\\ & =\left[\begin{array}{c} 0.01\sin(0.1t)+0.001\cos(0.5t)\\ 0.01\sin(0.1t)+0.001\cos(1.0t)\\ 0.01\sin(0.1t)+0.001\cos(1.5t) \end{array}\right] \end{array}$$

The noise magnitude in Equation (40) is chosen to be almost 0.01, and the signal to noise ratio is almost 20, such that the noise level in the numerical study is the same as that in the field tests [2].

**Figure 14:**
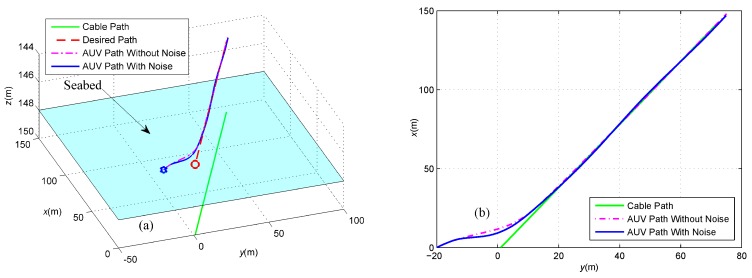
(**a**) Three-dimensional view of the cable tracking; and (**b**) Two-dimensional projection in the *X*-*Y* plane. These two pictures intuitively describe the cable tracking process, in which the green line represents the underwater cable, the red dashed line represents the desired tracking path, the magenta dot-dashed line represents the actual path of the under-actuated AUV without sensor noise and the blue line represents the actual path of the under-actuated AUV with sensor noise.

**Figure 15:**
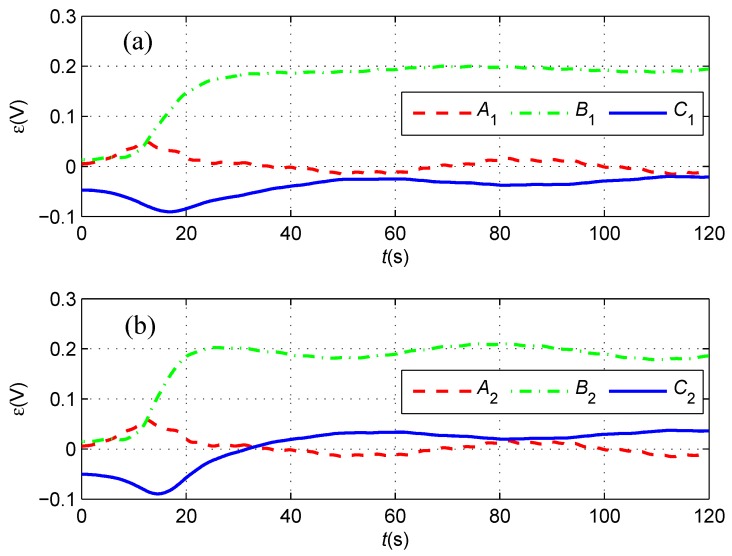
(**a**) The induced electromotive forces measured by the port tri-axial magnetometer; and (**b**) The induced electromotive forces measured by the starboard tri-axial magnetometer in the case of $k_{\varepsilon }=1$. The red dashed line, the green dash dotted line and the blue line represent the force components along the *x*-axis, *y*-axis and *z*-axis of the tri-axial magnetometer, respectively.

In this case, the blue cable tracking path is described in Figure 14, and the tracking path without sensor noise is depicted by a magenta dot-dashed line. Obviously, the under-actuated AUV also moves towards, converges to and finally follows the desired red path in the presence of sensor noise on the magnetometers. The transitions of the induced electromotive forces in the tracking period are described in Figure 15, where the force components along the *x*-axis and *y*-axis converge asymptotically and fluctuate slightly around zero and 0.19, respectively. In addition, the components along the *z*-axis have the same variation tendency, but a different direction at the final stages of tracking the cable, which indicates that the cable is broadly located below in the middle of two tri-axial magnetometers installed in the AUV.

**Figure 16:**
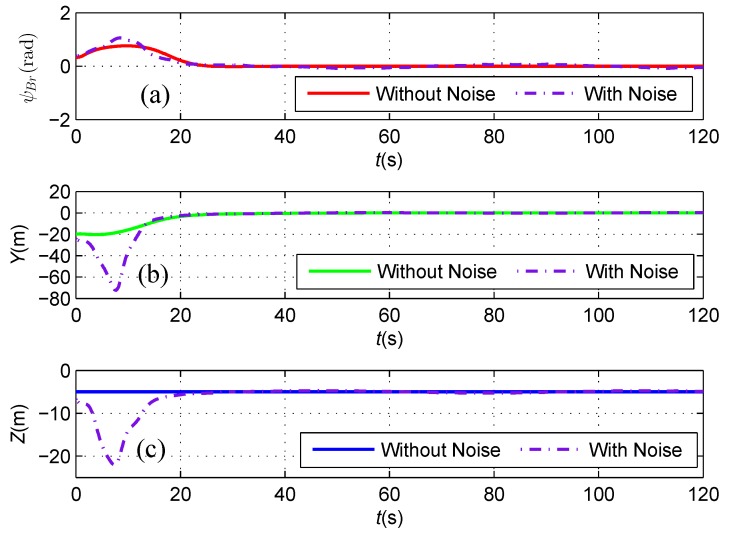
(**a**) The angle $\psi_{Br}$ between the AUV heading and the direction of the cable; (**b**) The horizontal offset *Y* between the AUV and the cable; and (**c**) The vertical distance *Z* between the AUV and the cable. Two simulation cases are plotted in the same figure, in which the solid line and dot-dashed line represent the error without noise and the error with noise, respectively.

**Figure 17:**
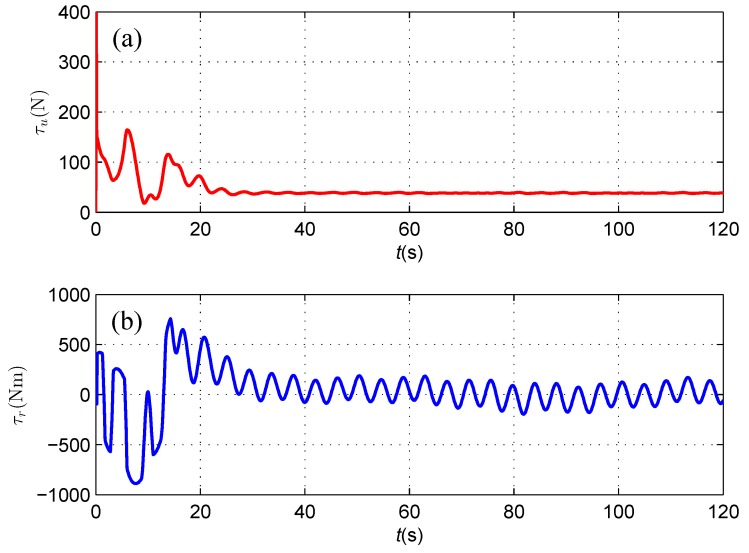
(**a**) Surge control force $\tau_{u}$; and (**b**) Yaw control moment $\tau_{r}$.

As shown in Figure 16, although a spike exists in the initial stage of measured horizontal distance and vertical distances from the magnetometer readings, which likely results from a relatively small signal to noise ratio when the AUV is away from the subsea cable, both the heading deviation between the direction of the cable and the AUV heading and the horizontal offset asymptotically reduce to zero, which illustrates that the AUV follows the cable route with its heading aligned with the orientation of the straight cable in the presence of sensor noise, and the algorithm proposed in this paper is proven to be robust against sensor noise in a suitable level. The available control inputs of the AUV including the surge force and yaw moment are depicted in Figure 17, where they keep changing against the position, orientation errors and sensor noise of the subsea cable tracking.

## 5. Conclusions

The inspection of the subsea cable route and the surviving seabed environment of the underwater communication cable is an essential and challenging task for the construction and maintenance of the underwater cable system. In this paper, a dedicated magnetic guidance and simplified control algorithm in the horizontal plane is proposed to enable the underwater cable to be precisely tracked by an inspection AUV without human beings in the loop. This work relies on a pair of tri-axial magnetic sensors and introduces a new analytic formulation to locate the orientation and the buried depth of the underwater cable. The simplified feedback linearizing controller is developed based on the magnetic guidance information to drive the 3-DOF AUV moving towards the desired path and inspecting along the underwater cable. Numerical simulation results show the effectiveness of the proposed magnetic guidance and control algorithm on the envisioned subsea cable tracking, which enables the potential protection of the seabed environment along the cable route.

As the AUV may suffer from unknown currents in the sea, future work will include the improvement of the controller to account for the presence of environmental disturbances. In addition, the seabed might have steep sand-waves, which motivates us to develop guidance and cable tracking algorithms in three-dimensional space that must make full use of the pitch angle and the vertical distance as an extension of the presented cable tracking in the fixed-depth plane.

## Figures and Tables

**Table 1:** The hydrodynamic parameters of the under-actuated AUV moving in the horizontal plane are obtained from Chapter 12 in [57].

Parameter	Value	Unit
*L*	1.5	m
$m_{11}$	1116	kg
$m_{22}$	2133	kg
$m_{33}$	4061	kg·m^2^
$d_{11}$	25.5	kg·s^−1^
$d_{22}$	138	kg·s^−1^
$d_{33}$	490	kg·m^2^·s^−1^
$d_{u2}$	0	kg·m^−1^
$d_{u3}$	0	kg·m^−2^·s
$d_{v2}$	920.1	kg·m^−1^
$d_{v3}$	750	kg·m^−2^·s
$d_{r2}$	0	kg·m^2^
$d_{r3}$	0	kg·m^2^·s

## Abbreviations

ROV	Remotely-operated vehicle
3-DOF	Three-degrees-of-freedom
AUV	Autonomous underwater vehicle
AC	Alternating current
AE	AQUA EXPLORER
LOS	Line-of-sight
ILOS	Integral line-of-sight
USV	Unmanned surface vessel
